# Serum levels of soluble urokinase plasminogen activator receptor in juvenile idiopathic arthritis: a single-center Swedish case-control study

**DOI:** 10.1186/s12969-023-00832-9

**Published:** 2023-05-29

**Authors:** Per Lewander, Lina Wirestam, Charlotte Dahle, Jonas Wetterö, Christopher Sjöwall

**Affiliations:** 1 Department of Paediatrics, Vrinnevi Hospital, Norrköping, Sweden; 2 Department of Biomedical and Clinical Sciences, Division of Inflammation and Infection/Rheumatology, Linköping University, SE–581 85 Linköping, Sweden; 3 Department of Biomedical and Clinical Sciences, Division of Inflammation and Infection/Clinical Immunology, Linköping University, Linköping, Sweden

**Keywords:** Soluble urokinase plasminogen activator receptor, Juvenile idiopathic arthritis, Joint damage, Biomarker

## Abstract

**Objectives:**

Reliable biomarkers in the early stages of idiopathic arthritis (JIA) are scarce and the disease heterogeneity makes it clinically challenging to predict the risk of joint damage. Biomarkers with prognostic potential are warranted in order to individualize treatment and follow-up in JIA. The soluble urokinase plasminogen activator receptor (suPAR) has been reported as an easily measurable biomarker for prognosis and severity in several rheumatic diseases but it has never been studied in JIA.

**Methods:**

Sera from 51 well-characterized patients with JIA and 50 age- and sex-matched control subjects were collected and stored for later analysis of suPAR. Patients were carefully followed clinically over 3 years and analysis of erythrocyte sedimentation rate, C-reactive protein, rheumatoid factor (RF) and antibodies against cyclic citrullinated peptides (anti-CCP) were analyzed as part of clinical routine. Signs of joint erosions were evaluated by radiography.

**Results:**

Overall, the levels of suPAR did not differ significantly between JIA patients and controls but those with polyarticular involvement showed higher suPAR (*p* = 0.013). In addition, elevated suPAR were associated with joint erosions (*p* = 0.026). Two RF/anti-CCP negative individuals with erosions showed high levels of suPAR.

**Conclusions:**

We present new data on the biomarker suPAR in JIA. Our results indicate that, apart from RF and anti-CCP, analysis of suPAR could be of additional value in assessing the risk of erosions. Analysis of suPAR early could potentially guide treatment decision-making in JIA, but our observations should be confirmed in prospective studies.

## Introduction

Juvenile idiopathic arthritis (JIA) constitutes a heterogeneous group of inflammatory joint disorders affecting children below the age of 16 years [1]. The etiology is unknown but, as in many other inflammatory disorders, environmental factors in combination with a genetic predisposition are believed to be of importance [2]. The disease course may be non-destructive and self-limiting, but up to 60% of the patients develop a chronic progressive disease associated with development of permanent joint damage [3]. According to a follow-up study of > 400 patients with JIA, long lasting (> 5 years) clinical remission off-treatment was found in 6% [3]. Patients with persistent oligoarticular JIA showed the best outcome with 68% remaining in clinical remission compared to 5% of rheumatoid factor (RF) positive patients [4]. A severe disease onset with persistent active disease, symmetrical joint involvement, early wrist or hip involvement, presence of RF and early signs of radiographic changes predict a more severe course. However, signs of tissue damage reflect that the inflammatory process has been ongoing for a considerable time [5, 6].

Highly effective disease-modifying antirheumatic drugs (DMARDs), including biological therapies, often downregulate the destructive inflammatory processes and should hence be initiated before tissue damage occurs [7, 8]. Unfortunately, at the time of diagnosis it is often difficult to predict the future risk for joint erosion and destruction. Since reliable predictive markers are largely lacking, the immunomodulatory treatments are usually introduced stepwise based on the clinical response over time [9, 10].

Biomarkers that associate with high, as well as low, risk of aggressive disease course are thus needed to individualize and optimize the treatment. The serum level of the soluble urokinase plasminogen activator receptor (suPAR; soluble CD87) is a promising candidate biomarker for prognosis and severity in several inflammatory and infectious diseases [11, 12]. suPAR is a part of the plasminogen activation system which has many functions in regulation of inflammation and tissue remodeling. The membrane-bound receptor (uPAR) is widely expressed on endothelial cells as well as different immune cell types and is upregulated by proinflammatory cytokines and growth factors [12]. In a previous cross-sectional study in cases with systemic lupus erythematosus (SLE), elevated levels of suPAR in plasma were shown to reflect damage accrual and also seemed valuable in predicting future irreversible organ damage in recent-onset SLE [11, 13]. In addition, levels of suPAR have been shown to correlate with disease activity in early rheumatoid arthritis and reflect accumulation of joint damage over time [14, 15].

Based on prior findings where suPAR has shown to associate with prognosis, and that suPAR has to our knowledge not previously been studied in JIA, we asked whether high suPAR levels are associated with erosions in JIA. In addition, we evaluated suPAR levels in relation to gender, age, disease duration, JIA subtypes, disease activity and presence of antinuclear antibodies (ANA), RF and antibodies against cyclic citrullinated peptides (anti-CCP).

## Methods

### Study populations

We included 51 children (32 girls and 19 boys), classified with JIA according to the International League of Associations for Rheumatology (ILAR) criteria [1]. Besides JIA subtypes, data regarding age, gender and antirheumatic treatments were recorded and the patients were carefully followed clinically over 3 years. Presence of joint swelling, tenderness or motion-induced joint pain without traumatic origin were evaluated according to the ACR definition of disease activity [4]. Radiography (X-ray) investigations of affected joints were performed as part of clinical routine in 47 (92%) patients. The X-rays were always evaluated at the same unit (Radiology Unit, Vrinnevi Hospital in Norrköping), but not necessarily by the same radiologist.

The control group comprised 50 sex- and age-matched children (31 girls and 19 boys) who lived in the same region as the patients, median age 13.3 years (range 2–18) and were recruited among friends (*n* = 36) and siblings (*n* = 14) to the patients. None of the controls had signs of arthritis or had a history of rheumatic disease, and neither of them had asthma, renal disease or hypertension. They were sampled once and not investigated by radiography.

Peripheral venous blood sampling was performed in patients and controls at inclusion. After centrifugation at 2000* g* for 10 min, sera were stored at − 70 °C until analysis of suPAR.

### suPAR analysis

For suPAR analysis, enzyme-linked immunosorbent assay (suPARnostic® AUTO Flex ELISA, ViroGates, Birkerod, Denmark) was performed according to manufacturer’s instructions [11]. Briefly, serum samples diluted 1:10 and peroxidase-conjugated anti-suPAR was mixed and then incubated in an anti-suPAR pre-coated 96-well plate. After 1 h incubation, tetramethylbenzidine substrate was added and the enzymatic reaction was stopped after 20 min by adding 2 N sulfuric acid and read at 450 nm (plate reader Sunrise, Tecan, Männedorf, Switzerland; software Magellan version 7.1, Tecan). All samples were run in duplicates.

A large Danish study of 5 538 adult individuals showed a mean serum concentration of suPAR of 3.51 ng/mL for men and 3.90 ng/mL for women [16]. The highest value among the controls herein (> 3.6 ng/mL) was used as cut-off; this corresponded to the 98th percentile among the healthy controls.

### ESR and CRP

Erythrocyte sedimentation rate (ESR; mm/h) and C-reactive protein (CRP; mg/L) were analyzed according to routine methods at the Department of Clinical Chemistry, Vrinnevi Hospital, Norrköping. CRP was analyzed by turbidimetry (Advia® 1800, Siemens Healthcare Diagnostics, Terrytown, NY, USA). Concentrations of CRP < 10 mg/L were reported as 5 mg/L.

### ANA

ANA were detected by indirect immunofluorescence (IF) microscopy using slides with fixed HEp-2 cells (Immunoconcepts, Sacramento, CA, USA) and fluorescein-isothiocyanate (FITC) conjugated γ-chain-specific anti-human IgG as detection antibody (Agilent, Glostrup, Denmark), as previously described [17]. The chosen cut-off for ANA corresponds to the 95th percentile among healthy blood donors (*n* = 300; 50% females, 50% males) in line with the international recommendations [18].

In addition, ANA-subspecificities (including SSA/Ro52 and SSA/R060, SSB/La, Sm, Sm/RNP, U1RNP, dsDNA, CENPB, Jo-1, Scl-70, PmScl, PCNA, histone and Ribosomal P were analyzed in all samples using addressable laser bead immunoassay (ALBIA) and the FIDIS™ Connective profile, Solonium software ver. 1.7.1.0 (Theradiag, Croissy-Beaubourg, France) at the Clinical immunology laboratory at University Hospital in Linköping [11].

### Anti-CCP and RF

Anti-CCP antibodies were analyzed, according to the manufacturer’s instructions, by fluorescence enzyme immunoassay (FEIA) on a Phadia 250 instrument with second generation cyclic citrullinated peptides (CCP) as antigen (EliA, Thermo Fischer AB, Uppsala, Sweden). RF was analyzed with nephelometric technique (Beckman Coulter, Inc., CA, USA) at the Department of Clinical Chemistry, University Hospital in Linköping.

### Statistics

Normal distribution was evaluated by using Shapiro-Wilk test and Kolmogorov-Smirnov test. Mann Whitney’s *U*-test was used to compare suPAR levels between JIA and controls as well as between oligoarticular, polyarticular, JPS, enthesitis-related arthritis, undifferentiated and controls. Possible correlations between suPAR and sex, age, disease duration or disease activity at the sampling occasion was evaluated by Pearson’s correlation. Pearson’s correlation was also used to analyze the levels of suPAR versus presence of RF and anti-CCP. Fisher’s exact test was used to analyze differences between high suPAR (> 3.6 ng/mL) among subjects with JIA compared to controls. Fisher’s exact test was also used to analyze possible associations between erosions and high suPAR levels (> 3.6 ng/mL), or RF positivity or anti-CCP positivity. P-values < 0.05 were considered statistically significant. Statistical analyses were performed using GraphPad Prism version 9 (GraphPad software Inc, La Jolla, CA, USA).

### Ethics approval

Oral and written informed consents were obtained from all patients and controls. The study was conducted according to the Declaration of Helsinki and the study protocol was approved by the Regional Ethics Boards regarding SLE (Linköping M70 − 07/2007).

## Results

### Patients

The JIA subtypes of the included patients were represented by oligoarticular arthritis (*n* = 21), polyarticular RF positive (*n* = 4) and RF negative (*n* = 13), juvenile psoriatic arthritis (JPS; *n* = 5), enthesitis-related arthritis (*n* = 4) and undifferentiated arthritis (*n* = 4). None was diagnosed with systemic JIA. Enrolment took place at planned outpatient visits to one pediatrician (P.L.) at the Department of Pediatrics, Vrinnevi Hospital, Norrköping during 2007–2013. At inclusion the disease duration varied between 0.1 and 13 years, with a median of 2.5 years. By using the ACR definition of disease activity, 46 subjects (90%) were identified as having ‘active disease’ at enrolment and 10 of them were newly diagnosed (< 6 months). Patient characteristics are detailed in Table 1.

**Table 1:** Characteristics and demographics of the included patients with JIA and the controls

		JIA (*n* = 51)	Controls (*n* = 50)
		*Median (range) or number (%)*	
**Age at enrolment**, ***years***		12.9 (1.6–17.9)	13.3 (2.0–17.9)
**Female sex**, ***n***		32 (62.7)	31 (62)
**JIA subtype**, ***n***	Oligoarticular	21 (41.2)	N/A
	Polyarticular RF+	4 (7.8)	N/A
	Polyarticular RF-	13 (25.5)	N/A
	JPS	5 (9.8)	N/A
	Enthesitis-related arthritis	4 (7.8)	N/A
	Undifferentiated	4 (7.8)	N/A
**Disease duration at inclusion**, ***months***		19 (0 − 161)	
**Active disease**, ***n***		46 (90.2)	
**C-reactive protein**, ***mg/L***		9 (5–40)	
**Erythrocyte sedimentation rate**, ***mm/h***		10 (2–34)	
**ANA+**, ***n***		22 (43.1)	
**RF+**, ***n***		5 (9.8)	
**Anti-CCP+**, ***n***		6 (11.8)	
**RF+/anti-CCP+**, ***n***		4 (7.8)	
**NSAID**, ***n***		28 (54.9)	
**Oral steroids**, ***n***		16 (31.3)	
**Intraarticular steroids**, ***n***		32 (62.7)	
**Conventional synthetic DMARDs**, ***n***		27 (52.9)	
	Methotrexate	23 (45.1)	
	Sulfasalazine	4 (7.8)	
**Biological DMARDs**, ***n***		8 (15.7)	
	Adalimumab	4 (7.8)	
	Etanercept	4 (7.8)	

*ANA* Antinuclear antibodies, *anti-CCP* Anti-cyclic citrullinated peptide antibody, *DMARDs* Disease Modifying Anti-Reumatic Drugs, *JIA* Juvenile idiopathic arthritis, *JPS* Juvenile psoriatic arthritis, *N/A* Not applicable, *NSAID* Non-steroid antiinflammatory drugs, *RF* Rheumatoid factor

### Radiography

Skeletal X-ray investigations of affected joints were performed 5–204 months after disease onset in 47 patients. Depending on symptoms, some children performed multiple X-rays. Signs of erosions were found in 15 individuals (31.9%). One patient was lost to follow-up because of migration and one patient declined the radiography investigation. Two subjects had very mild disease where investigation with X-ray was judged clinically not motivated. In fact, 3 out of the 15 children with erosions had signs of erosive disease at inclusion and the remaining 12 developed erosions during follow-up. Detailed information on the 15 children with erosive disease is given in Table 2.

**Table 2:** Detailed characteristics of the 15 patients with joint erosions

Number	Sex (F/M)	Age at inclusion (years)	JIA subtype	Anti-CCP (pos/neg)	RF (pos/neg)	suPAR level (ng/mL)	Ongoing medication	Erosions detected (time from inclusion; months)
1	F	17.8	Polyarticular	pos	neg	2.97	Prednisolone, MTX, NSAID, i.a. corticosteroids	58
2	M	15.0	Enthesitis-related arthritis	neg	neg	5.05	Prednisolone, MTX, i.a. corticosteroids	31
3	F	12.8	Polyarticular	pos	pos	4.88	MTX, NSAID, i.a. corticosteroids	19
4	M	15.3	Oligoarticular	pos	pos	3.04	NSAID	20
5	M	16.5	JPS	neg	neg	1.57	Sulfasalazine, NSAID	4
6	M	17.7	Enthesitis-related arthritis	neg	neg	3.02	i.a. corticosteroids	11
7	F	15.0	Undifferentiated	neg	neg	1.95	‒	47
8	M	15.3	Enthesitis-related arthritis	neg	neg	2.65	Sulfasalazine, NSAID	-1
9	F	13.4	Polyarticular	neg	neg	2.95	Prednisolone, MTX, Etanercept	38
10	F	14.7	Oligoarticular	neg	neg	2.48	NSAID, i.a. corticosteroids	-16
11	F	14.8	Polyarticular	pos	pos	9.74	Prednisolone, MTX, i.a. corticosteroids	40
12	F	8.3	Polyarticular	neg	pos	3.70	Prednisolone, MTX, Adalimumab	39
13	F	16.5	Polyarticular	neg	neg	3.33	Prednisolone, MTX, i.a. corticosteroids	13
14	M	14.0	Enthesitis-related arthritis	neg	neg	2.83	Prednisolone, Sulfasalazine, Adalimumab	28
15	M	14.9	JPS	neg	neg	2.12	Prednisolone, MTX, NSAID	-1

*Anti-CCP* Anti-cyclic citrullinated peptide antibody, *i.a* Intraarticular, *JIA* Juvenile idiopathic arthritis, *JPS* Juvenile psoriatic arthritis, *MTX* Methotrexate, *NSAID* Non-steroid antiinflammatory drugs, *RF* Rheumatoid factor, *suPAR* Soluble urokinase plasminogen activator receptor

### suPAR

**Fig. 1:**
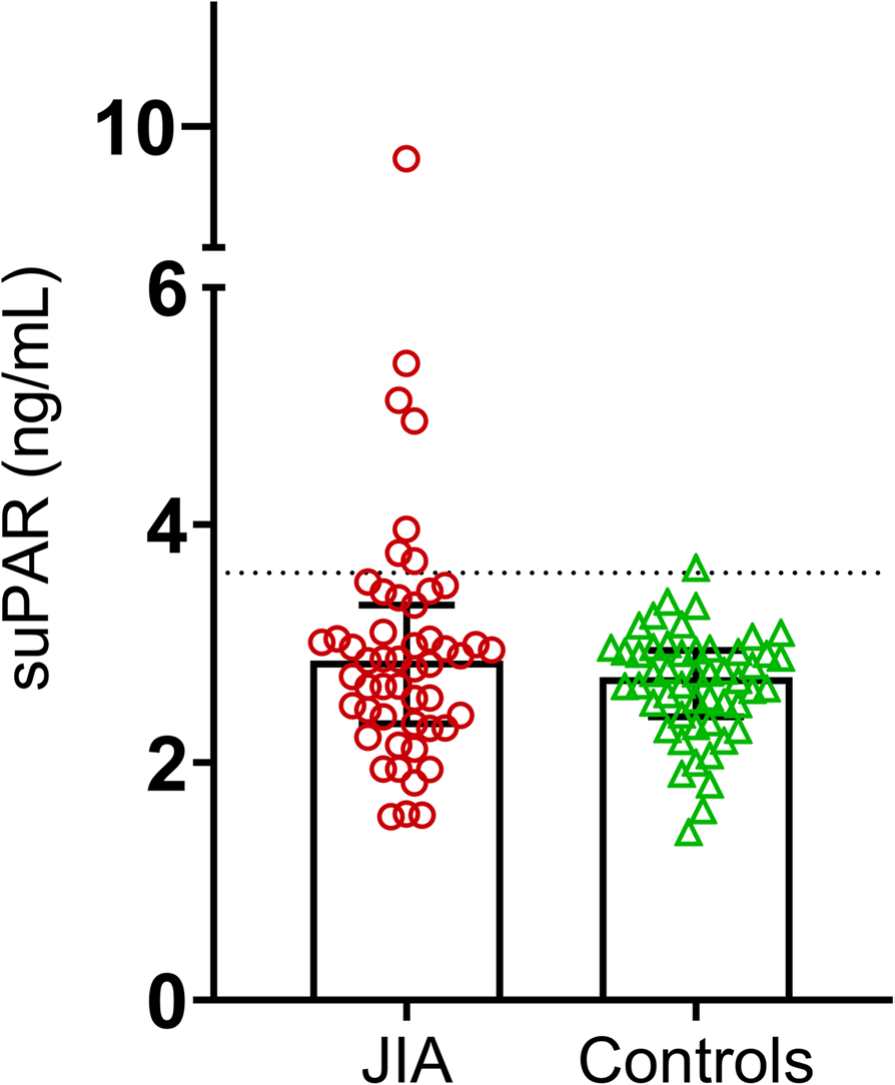
Serum concentrations of suPAR shown with median ± interquartile range (IQR) in the group of patients with JIA (*n* = 51) as well as in healthy sex- and age-matched controls (*n* = 50). The dotted line represents the cut-off, which was based on the 98th percentile of the controls

**Fig. 2:**
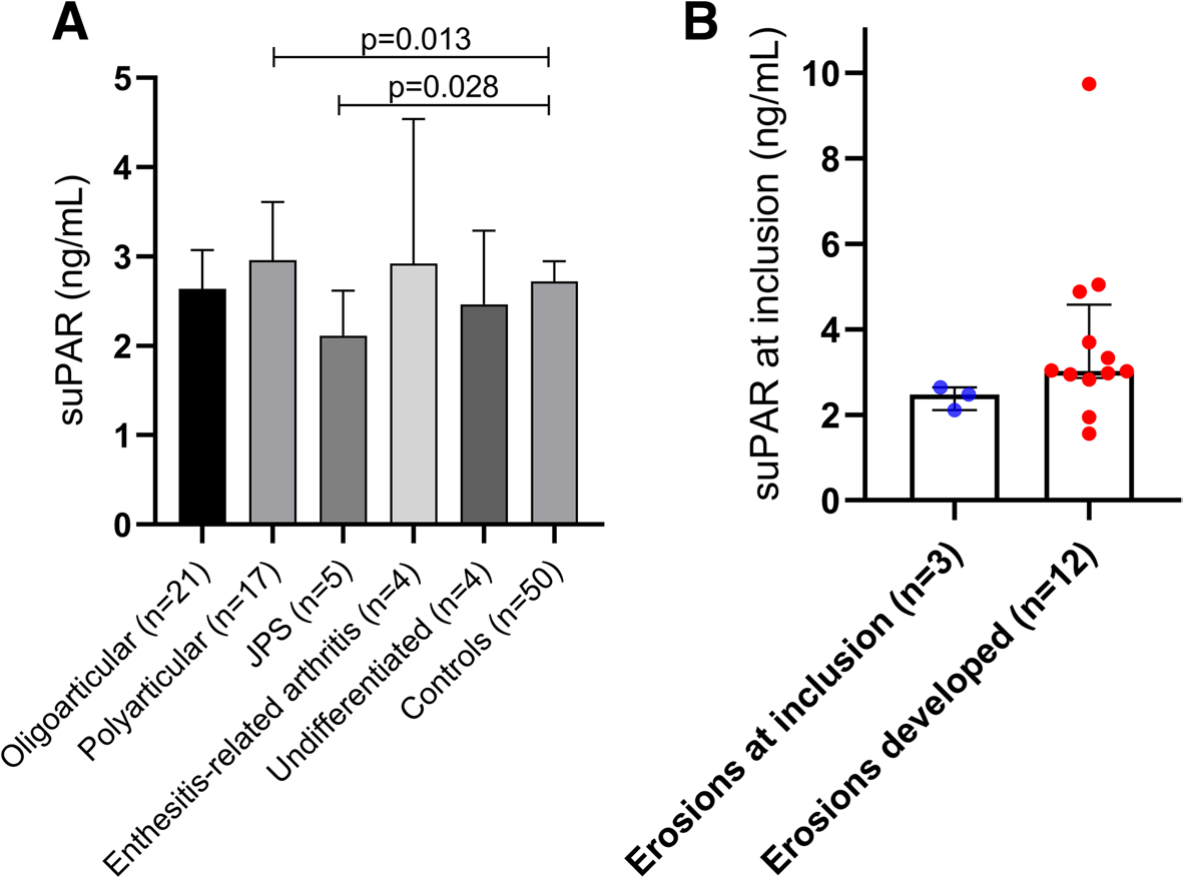
**A** Serum levels of suPAR with median ± interquartile range (IQR) in the different subgroups of JIA and in healthy sex- and age-matched controls. Polyarticular JIA showed significantly higher levels of suPAR than controls. In contrast, suPAR levels in JPS were lower than in controls. **B** suPAR concentration shown with median ± IQR in children with erosions at baseline versus those who developed erosions during follow-up. All comparisons used Mann-Whitney *U* test

In total, as demonstrated in Fig. 1, the median levels of circulating suPAR did not differ significantly between JIA and controls (2.86 ng/mL vs. 2.72 ng/mL). Neither were the levels of suPAR associated with sex, age, disease duration or disease activity at the sampling occasion. However, compared to controls, the median level of suPAR was significantly higher in the polyarticular subgroup (*n* = 17; *p* = 0.013) and lower in patients with JPS (*n* = 5, *p* = 0.028) as shown in Fig. 2A.

Levels of suPAR above the cut-off (> 3.6 ng/mL) was significantly more often found among subjects with JIA compared to controls (7/51 versus 1/50; *p* = 0.029). The control subject with suPAR level 3.67 ng/mL (above cut-off) was a friend, not a sibling. Patients with elevated suPAR were more prone to present with current or future joint damage; 5 of 7 patients (71%) showing suPAR concentration > 3.6 ng/mL had signs of, or developed, bone or cartilage damage within 3 years from baseline compared to 10 of 40 (25%) patients with suPAR concentration < 3.6 ng/mL (*p* = 0.026). All four patients with suPAR levels > 4 ng/mL had signs of erosions. As demonstrated in Fig. 2B, there was a trend of higher suPAR among patients that developed erosions during follow-up than in those with erosions at baseline (*p* = 0.10). Liver enzymes were checked regularly as part of clinical routine. None of the children with erosions showed elevation of alanine transaminase levels. The subtypes of JIA with suPAR levels > 3.6 ng/mL included the polyarticular (*n* = 4), the oligoarticular (*n* = 2) and the enthesitis-related arthritis (*n* = 1) variants.

**Fig. 3:**
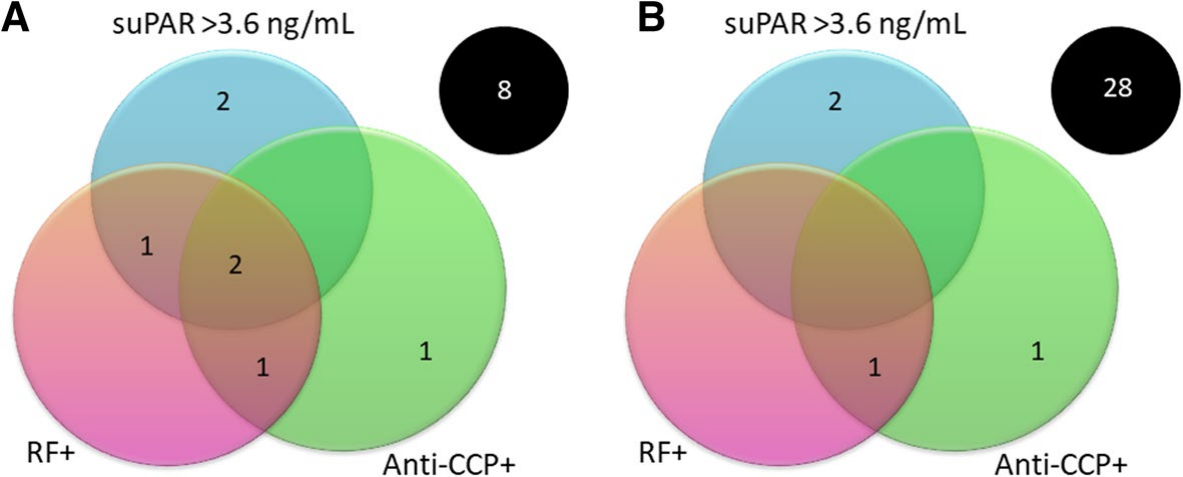
Venn diagrams illustrating the prevalence of rheumatoid factor (RF), anti-cyclic citrullinated peptide antibodies (anti-CCP) and suPAR levels > 3.6 ng/mL among (**A**) the 15 patients with erosions found on radiography and (**B**) the 32 subjects without joint erosions. The black circles indicate patients who were negative for RF and anti-CCP and had a serum concentration of suPAR < 3.6 ng/mL

Figure 3 illustrates the prevalence of RF, anti-CCP, and suPAR levels > 3.6 ng/mL among (A) patients with erosions and (B) patients without erosions. RF and anti-CCP positivity were strongly associated with joint erosions (*p* = 0.001 and p = 0.008, respectively; Fig. 3A). Two patients with signs of joint erosions were negative for both RF and anti-CCP but showed levels of suPAR > 3.6 ng/mL (Fig. 3A).

We observed no significant association between the levels of suPAR and presence of RF and/or anti-CCP. In addition, neither ESR (*p* = 0.30) nor CRP (*p* = 0.87) levels were significantly correlated with suPAR levels.

## Discussion

To our knowledge, this study is the first to evaluate the role for circulating levels of suPAR as a prognostic biomarker in JIA. We observed that elevated levels of suPAR were associated with increased risk of joint erosions. In our hands, elevated suPAR levels associated with joint damage in some patients with JIA even in the absence of concomitant RF or anti-CCP. Our data needs to be confirmed by others but could potentially be important and useful in guiding treatment decision-making early in patients with JIA. The findings are in line with previous observations in adult RA and SLE where levels of suPAR were shown to associate with established or future development of tissue damage and/or prognostics [11, 15].

Overall, no difference of the suPAR levels between JIA and controls were found at group level. Yet, importantly, the proportion of individuals with radiological signs of joint destruction were higher in the subgroup of JIA with suPAR levels above cut-off out of which some lacked the established JIA damage risk factors RF and anti-CCP. This finding supports that suPAR can be a valuable prognostic marker that potentially could be included in the primary evaluation when JIA is diagnosed, and treatment strategy is planned.

In our study population, 15 out of the 47 patients that underwent investigation with X-ray already had or developed radiological signs of joint erosion within three years after inclusion in the study. The increased levels of suPAR were predominantly detected in polyarticular JIA but can be a result of few individuals in the other subgroups. Reliable predictive markers of aggressive disease course are important for identifying the individuals in need of more efficacious treatments before irreversible joint destruction occur, but it is also important to avoid treating patients with lower risk [9]. Although the results indicate that analysis of suPAR could be of additional value in assessing the risk of erosions in JIA, the observations need to be confirmed in prospective studies. Such studies should also include a number of additional variables, including number of active joints and patient-reported outcome measures.

Our study has several limitations. Obviously, the number of patients and the wide spectrum of disease duration constitute limitations. The clinical heterogeneity of JIA makes it difficult to draw firm conclusions of suPAR levels in relation to different JIA subtypes. In addition, the background medication (including use of corticosteroids) was different, and we cannot exclude that this could have affected the results. Furthermore, no reference interval for suPAR is available for children and thus a larger control population would have been beneficial. However, there is only one previous article investigating suPAR in a pediatric study population and that study did not include any controls [19]. In addition, our study has several major strengths. The Swedish healthcare is public, tax-funded and offers universal access which excludes risks of selection bias. It was a single investigator study with only one pediatrician examining the patients over a long period of time. The suPAR analyses were performed at the same time in patients and in the well-matched controls with the same batches, and at the same single accredited laboratory.

## Conclusions

To conclude, our observations indicate that, in patients with JIA, analyses of suPAR in circulation could be of additional value as a biomarker associated with joint erosions. The data support that analysis of suPAR could be useful in patients testing negative for RF and anti-CCP. Potentially, suPAR could be used in guiding treatment decision-making early in JIA to avoid joint destruction in the future. However, our findings call for confirmation before firm conclusions can be drawn.

## Data Availability

The datasets used and/or analyzed during the current study are available from the corresponding author on reasonable request.

## Abbreviations

CRP: C-reactive protein
ESR: Erythrocyte sedimentation rate
RF: Rheumatoid factor
Anti-CCP: Anti-cyclic citrullinated peptide antibody
ANA: Antinuclear antibodies
JIA: Juvenile idiopathic arthritis
JPS: Juvenile psoriatic arthritis
DMARDs: Disease-modifying anti-rheumatic drugs
NSAID: Non-steroid anti-inflammatory drugs
